# The Bloom-Forming Dinoflagellate *Karenia mikimotoi* Adopts Different Growth Modes When Exposed to Short or Long Period of Seawater Acidification

**DOI:** 10.3390/toxins13090629

**Published:** 2021-09-08

**Authors:** Yuanyuan Li, Zhengli Zhou, Yijun Li, Yanqun Wang, Mengxue Xu, Bin Zhou, Keyu Lu, You Wang

**Affiliations:** 1 Department of Marine Ecology, College of Marine Life Science, Ocean University of China, Qingdao 266003, China; liyuanyuan1004@stu.ouc.edu.cn (Y.L.); wangyanqun2016@stu.ouc.edu.cn (Y.W.); xumengxue@stu.ouc.edu.cn (M.X.); zhoubin@ouc.edu.cn (B.Z.); ucfalux@ucl.ac.uk (K.L.); 2 Pilot Laboratory for Marine Ecology and Environmental Science, Qingdao National Laboratory for Marine Science and Technology, Qingdao 266071, China; 3 College of Plant Science, Tarim University, Alar 843300, China; zzlzkytd@163.com; 4 Institute of Biomedical Engineering, College of Life Sciences, Qingdao University, Qingdao 266071, China; 2019025187@qdu.edu.cn

**Keywords:** seawater acidification, *Karenia mikimotoi*, apoptosis, cell cycle, photosynthetic carbon fixation, growth modes

## Abstract

Impacts of ocean acidification (OA) on noncalcifying organisms and the possibly responsible mechanism have aroused great research interests with the intensification of global warming. The present study focused on a noxious, noncalcifying, bloom-forming dinoflagellate, *Karenia mikimotoi* (*K. mikimotoi*), and its variation of growth patterns exposed to different periods of seawater acidification with stressing gradients was discussed. The dinoflagellates under short-time acidifying stress (2d) with different levels of CO_2_ presented significant growth inhibition (*p* < 0.05). The cell cycle was obviously inhibited at S phase, and the photosynthetic carbon fixation was also greatly suppressed (*p* < 0.05). Apoptosis was observed and the apoptotic rate increased with the increment of *p*CO_2_. Similar tendencies were observed in the key components of mitochondrial apoptotic pathway (the mitochondrial membrane potential (MMP), Caspase-3 and -9, and Bax/Bcl-2 ratio). However, under prolonged stressing time (8 d and 15 d), the growth of dinoflagellates was recovered or even stimulated, the photosynthetic carbon fixation was significantly increased (*p* < 0.05), the cell cycle of division presented little difference with those in the control, and no apoptosis was observed (*p* > 0.05). Besides, acidification adjusted by HCl addition and CO_2_ enrichment resulted in different growth performances, while the latter had a more negative impact. The results of present study indicated that (1) the short-time exposure to acidified seawater led to reduced growth performance via inducing apoptosis, blocking of cell cycle, and the alteration in photosynthetic carbon fixation. (2) *K. mikimotoi* had undergone adaptive changes under long-term exposure to CO_2_ induced seawater acidification. This further demonstrated that *K. mikimotoi* has strong adaptability in the face of seawater acidification, and this may be one of the reasons for the frequent outbreak of red tide. (3) Ions that dissociated by the dissolved CO_2_, instead of H^+^ itself, were more important for the impacts induced by the acidification. This work thus provides a new perspective and a possible explanation for the dominance of *K. mikimotoi* during the occurrence of HABs.

## 1. Introduction

Industrialization and fossil fuel combustion have increased the atmospheric CO_2_ concentration from a preindustrial level of approximately 280 ppmv (pH 8.2) to the current level of approximately 390 ppmv (pH 8.1) [1,2], and the excessive uptake of anthropogenic CO_2_ from the atmosphere subsequently results in ocean acidification (OA) [2,3]. The current prediction suggests the atmospheric CO_2_ concentrations would increase to 1000 ppmv and 2000 ppmv, respectively, by the years 2100 and 2300 if the present energy utilization structure persists [3], which is predicted to cause the decrease of ocean pH levels by as much as 0.3~0.4 units. OA would lead to substantial changes in ocean carbonate chemistry, decreasing the carbonate ion concentrations and elevating the bicarbonate ions [4,5].

The ecological impact of OA has aroused worldwide attention because it has been recognized as an important driver controlling the distribution, morphology, and biochemical performance of biological systems, which might ultimately result in changes in biodiversity, trophic interactions, and other ecosystem processes [6,7,8]. Generally, seawater acidification affects marine organisms through two pathways. First, it affects calcification rates by decreasing the calcium carbonate (CaCO_3_) saturation. This pathway usually occurs in calcifying organisms, and most studies have been devoted to these organisms [9,10,11,12,13]. Second, it causes disturbances in acid–base physiological processes, including acid–base regulation, oxygen transport, and metabolism, which has mainly been discussed in the context of noncalcifying organisms [2,12,14,15]. Marine microalgae are the base of marine ecosystems, and the recent research showed controversies about the effects of elevated CO_2_ levels on phytoplankton [16]. Some reports showed that elevated CO_2_ levels inhibited the microalgal growth mainly by influencing inorganic carbon (Ci) acquisition in phytoplankton [17,18,19,20,21]. However, neutral [22,23] or stimulative impacts [24,25] were also observed.

Harmful algal blooms (HABs), which are known for causing severe damage to the marine ecological environment and marine economy, as well as to public health, are increasing in frequency, magnitude, and duration worldwide [26]. Dinoflagellates are well known as the major HAB-causing species. *Karenia mikimotoi* is a toxic and noncalcifying bloom-forming dinoflagellate. It has been well documented that *K. mikimotoi* exerts detrimental impacts, including lethality, on a wide range of marine life, including marine fauna [27,28] and seaweeds [29]. Three kinds of toxins were speculated to be involved in *K. mikimotoi*-induced toxicity: hemolytic toxins [30], cytotoxins [31], and reactive oxygen species (ROS) [32,33]. Previous studies have suggested that hemolytic toxin was the main cause of the large number of marine organism deaths caused by the red tide of *K. mikimotoi*. Direct contact with living *K. mikimotoi* cells was the main way to cause zooplankton death. The hemolytic toxin and fish toxin produced by *K. mikimotoi* can dissolve the gill tissue of fish, and even cause the death of fish [30]. Previous studies have also found that *K. mikimotoi* can generate reactive oxygen species (ROS). *K. mikimotoi* may exhibit a toxic effect on marine organisms through ROS [32,33], but the role of ROS in the toxic mechanism of *K. mikimotoi* remains unclear. The latest reports have suggested that seawater acidification have an impact on the bloom type, extent, and duration because of the different responses of potentially dominant species and inferior species [34]. Elucidating the different performances of bloom-forming microalgae under acidification is of great concern. However, information on the different growth performance and the adaptive mechanism of noncalcifying microalgae with acidifying exposure remains scarce [35,36,37]. Although recent research has demonstrated the linkage between seawater acidification and bloom occurrence [38,39,40], such works often failed to interpret the possible mechanisms attributing to this linkage, which is expected to be resolved by this study. Therefore, the growth performances of *K. mikimotoi* exposed to different acidification levels lasting for different durations were analyzed, and the possible adaptive mechanism involved in this process was discussed. The present study would provide a new perspective and a possible explanation for the dominance of *K. mikimotoi* during the occurrence of HABs regarding physiological adaption to seawater acidification.

## 2. Results

Different growth modes of microalgae exposed to short or long period of seawater acidification were explored from three aspects, namely photosynthetic carbon fixation, that was extremely sensitive to changes in CO_2_ concentration, as well as apoptosis and cell cycle, which were closely related to cell proliferation.

### 2.1. Changes of Population Dynamics to Different pCO_2_ Levels

**Figure 1:**
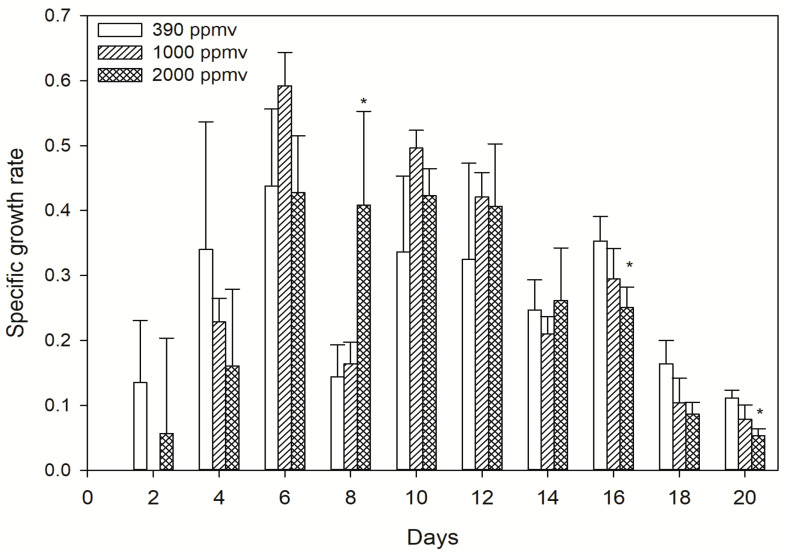
The specific growth rate of *K. mikimotoi* exposed to different *p*CO_2_ levels. * indicated the significant difference between the treatment groups with the control group at the *p* < 0.05 level, and data were expressed as means ± SD (*n* = 3).

The growth of *K. mikimotoi* in different *p*CO_2_ treatments is shown in Figure 1. The specific growth rate (*μ*) was inhibited in both treatment groups during the first four days after inoculation, while a relatively higher one was found thereafter compared with that of the control. A logistic equation was applied to simulate the growth performance, and the results showed the carrying capacity (*K*) as well as the maximum instantaneous growth rate (*r*) in either treatment groups to be higher than those in the control. However, the smaller *Tp* (the inflection point of the population growth curve) of the treatment groups may be related to the significantly increase in growth rate which made the population enter the stationary phase faster (Table 1). It seems that short-term acidification stress inhibited the population increase within a certain range, but relatively long-term (4~14 days in the present study) acidification stress stimulated growth. This observation differs with the documented results on microalgae exposed to environmental stress and carbon-related processes because the specificity of CO_2_ treatment was speculated to provide more evidence.

### 2.2. Alteration in the Photosynthetic Carbon Fixation of K. mikimotoi Exposed to Different pCO_2_ Levels

**Figure 2:**
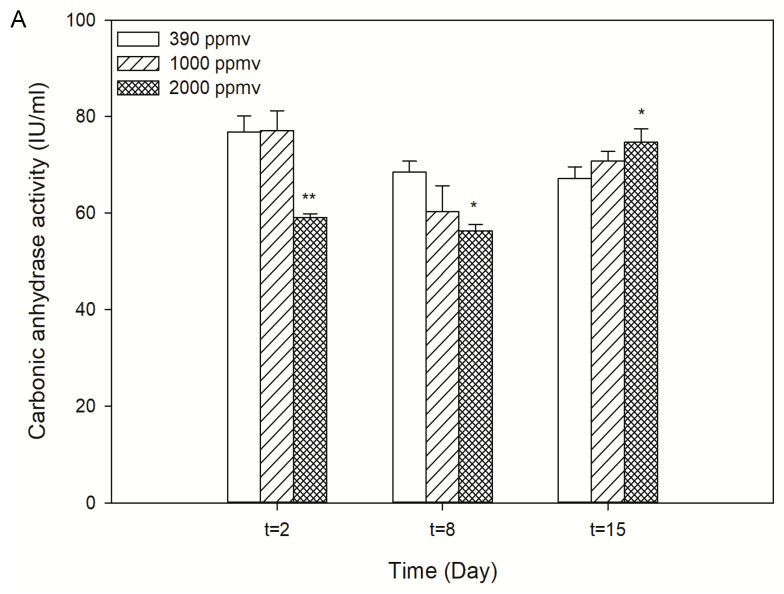

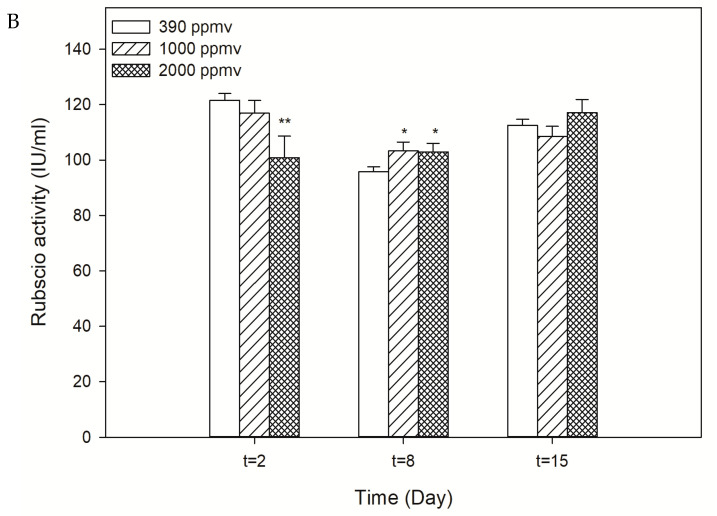
(**A**) Carbonic anhydrase activity of *K. mikimotoi* exposed to different *p*CO_2_ levels; (**B**) Rubisco activity of *K. mikimoto*i exposed to different *p*CO_2_ levels. * indicated the significant difference between the treatment groups with the control group at *p* < 0.05 level and ** indicated the most significant at *p* < 0.01 level, the data are shown as the mean ± SE (*n* = 3).

The increase in *p*CO_2_ decreased the activity of CA during the first eight days after exposure, and the most obvious inhibition was found in the 2000 ppmv treatment group compared with that of the control (*p* < 0.01). However, the opposite occurred after 15 days of exposure. The CA activity of the 2000 ppmv group was significantly increased compared to the control (*p* < 0.05) (Figure 2A). Different tendencies were observed in the changes of Rubisco activity. In fact, a clear time-dependent trend was observed in the 2000 ppmv treatment group, and the elevated *p*CO_2_ significantly decreased the activity of Rubisco in *K. mikimotoi* at 24 h after exposure. Rubisco activity increased steadily over time from the day 8 and reached its peak at the end of the experiment. Only a slight difference was observed in the 1000 ppmv group compared with the control group during the whole treatment (Figure 2B). Short-term exposure to seawater acidification inhibited the photosynthetic carbon fixation ability of *K. mikimotoi*, but with long-term exposure, the photosynthetic carbon fixation ability could recover and even improve. As a result, there was a consistency between the population dynamics and photosynthetic carbon fixation, and both changed with acidification time. The alteration in photosynthetic carbon fixation may affect the energy distribution of *K. mikimotoi* and then the population dynamics. This result provided a possible explanation for the changes in the population dynamics.

### 2.3. Alteration of Cellular Apoptosis of K. mikimotoi When Exposed to Different pCO_2_ Levels

**Figure 3:**
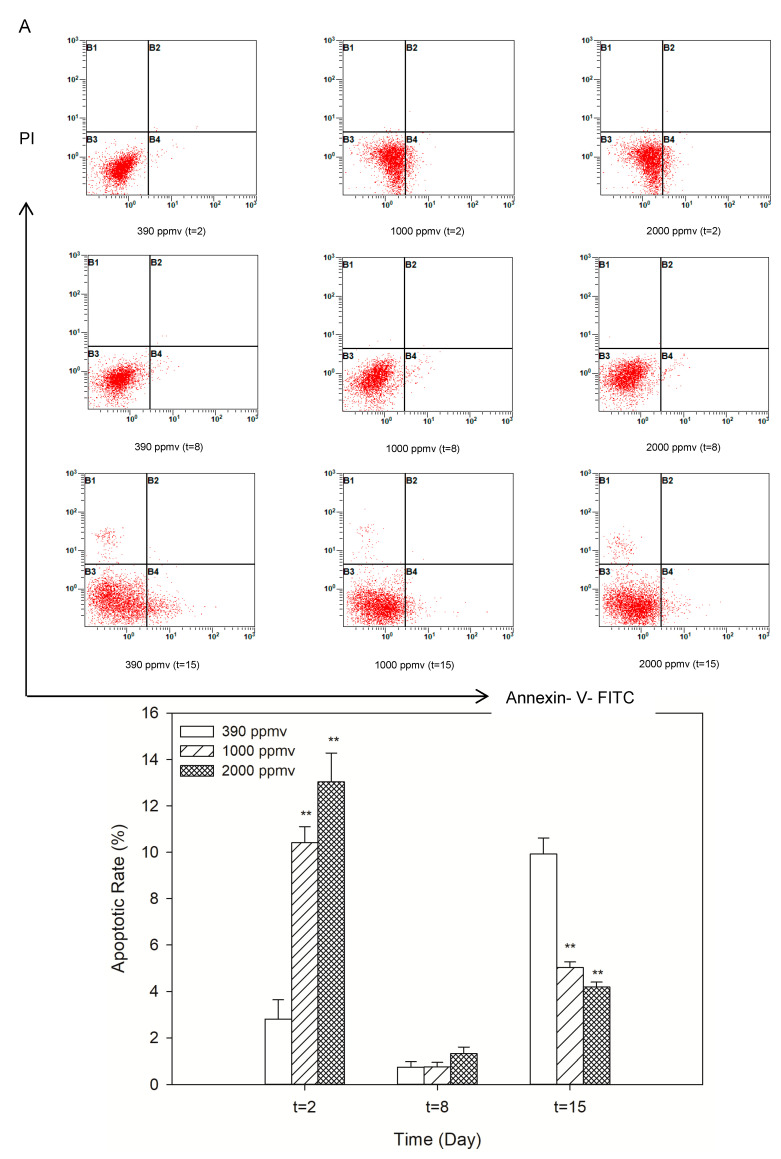

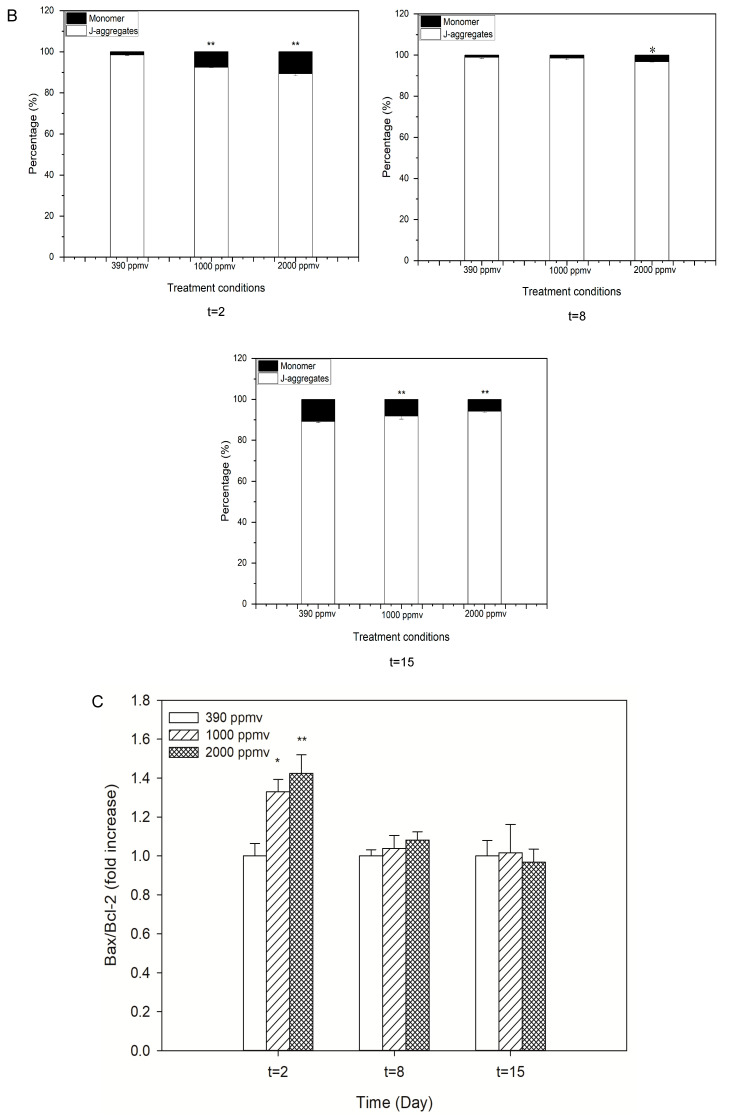

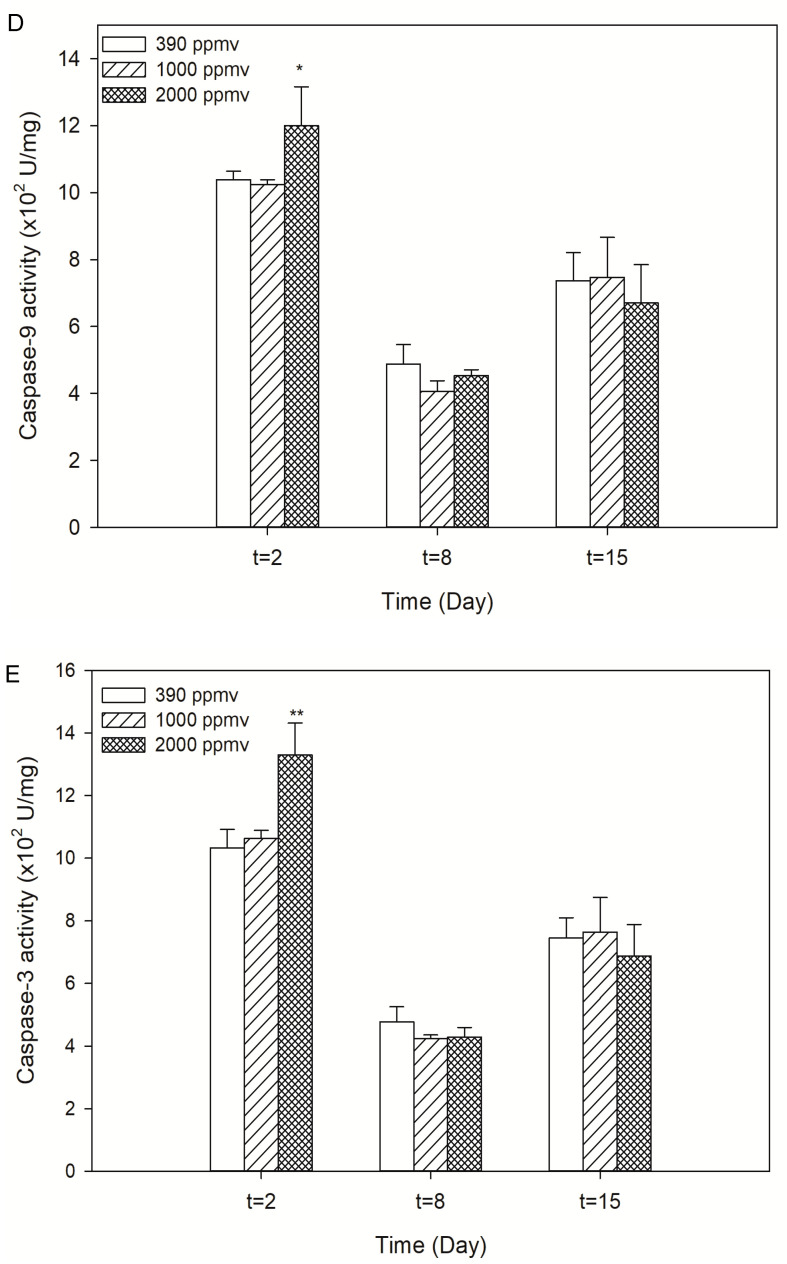
Alteration of cellular apoptosis of *K. mikimotoi* when exposed to different *p*CO_2_ levels. (**A**) Representative graphs of cell apoptosis obtained from flow cytometry analysis and the percentage of apoptotic cells were shown by histogram. (**B**) changes of mitochondirol membrane potential (MMP): percentage of JC-1 with monomer and J-aggregate were shown by histogram; (**C**) changes of Bax/Bcl-2 ratio; (**D**) changes of Caspase-9 activity; (**E**) changes of Caspase-3 activity. * indicated the significant difference between the treatment groups with the control group at *p* < 0.05 level and ** indicated the most significant at *p* < 0.01 level, and data were expressed as means ± SD (*n* = 3). Note: the damaging level indicated from the percentage of JC-1 monomer with green fluorescence.

Elevated *p*CO_2_ induced cellular apoptosis of *K. mikimotoi* at 24 h after exposure, and the apoptotic rate greatly increased in a concentration-dependent manner from approximately 2.8% in the control to 10.41% and 13.03% in the 1000 ppmv and 2000 ppmv groups, respectively (Figure 3A). Statistical significance was observed between the treatment groups and the control (*p* < 0.05). However, the apoptotic rate in the 1000 ppmv and 2000 ppmv treatment groups almost decreased to zero on the eighth day after treatment, which was only slightly different from that in the control. When the population grew to approximately the plateau phase, the cells began to undergo apoptosis again. At this time, the apoptosis rate of the 390 ppmv group (i.e., the control group) was higher than 1000 ppmv and 2000 ppmv groups.

To further support the abovementioned observation, a series of indices closely related to the apoptotic pathway was analyzed. The trend of the change in the percentage of JC-1 monomer was somewhat similar to that of the apoptotic rate (Figure 3B). The percentage of cells in the JC-1 monomer that indicated mitochondrial membrane potential (MMP) damage was greatly increased, by approximately 5.96% and 9.17% in the 1000 ppmv and 2000 ppmv groups, respectively, after 24 h of exposure to elevated *p*CO_2_. Similarly, the JC-1 monomer in the 1000 ppmv and 2000 ppmv groups was almost undetectable on the eighth day after treatment, which was not significantly different from the results of the control group. However, when the population grew to approximately the plateau phase, the JC-1 monomer in the experimental group increased significantly, and the percentage of JC-1 monomer at 1000 ppmv and 2000 ppmv was approximately 2.67% and 5.03% lower than the percentage of JC-1 monomer at 390 ppmv, respectively.

In addition, the expression levels of Bcl-2 and Bax were analyzed by ELISA, and the ratio of Bax/Bcl-2 was estimated (Figure 3C). The results showed that the 1000 ppmv and 2000 ppmv groups, which had ratios approximately 1.33- and 1.42-fold higher than that of the 390 ppmv group in the first two days after exposure, were highly significant. However, as the acidification time increased, seawater acidification caused by 1000 ppmv and 2000 ppmv had little effect on the Bax/Bcl-2 ratio. There was almost no difference in the ratio of Bax/Bcl-2 between the treatment groups and the control group, and the ratio of each group was close to 1 after exposure for eight days.

The key components of the apoptosis pathway, i.e., Caspase-9 and Caspase-3, were examined. This examination consistently showed that both CO_2_ concentration and acidification time were associated with the Caspase-3 and -9 activities of *K. mikimotoi*. On the second day, the activity of Caspase-9 in the treatment groups steadily increased as the CO_2_ concentration increased and reached a peak at 2000 ppmv, which was approximately 1.15-fold higher than that in the control group (Figure 3D). However, as the acidification time increased, the Caspase-9 activity of the treatment groups significantly decreased, and after eight days of exposure there was almost no difference in Caspase-9 activity between the treatment and the control groups. A similar trend was observed for Caspase-3, which increased by approximately 1.29-fold (*p* < 0.05) (Figure 3E) in the 2000 ppmv group compared with that in the control group on the second day. As the acidification time increased, the Caspase-3 activity in the 1000 ppmv and 2000 ppmv treatment groups significantly decreased, which presented little difference with that in the control.

The above results indicated that short-term (i.e., 24 h) elevated *p*CO_2_ induced the apoptosis of *K. mikimotoi*. However, with prolonged exposure, the effect of elevated *p*CO_2_ on the apoptosis of *K. mikimotoi* gradually weakened or even disappeared.

### 2.4. Alteration of the Cell Cycle of K. mikimotoi Due to Elevated pCO_2_

**Figure 4:**
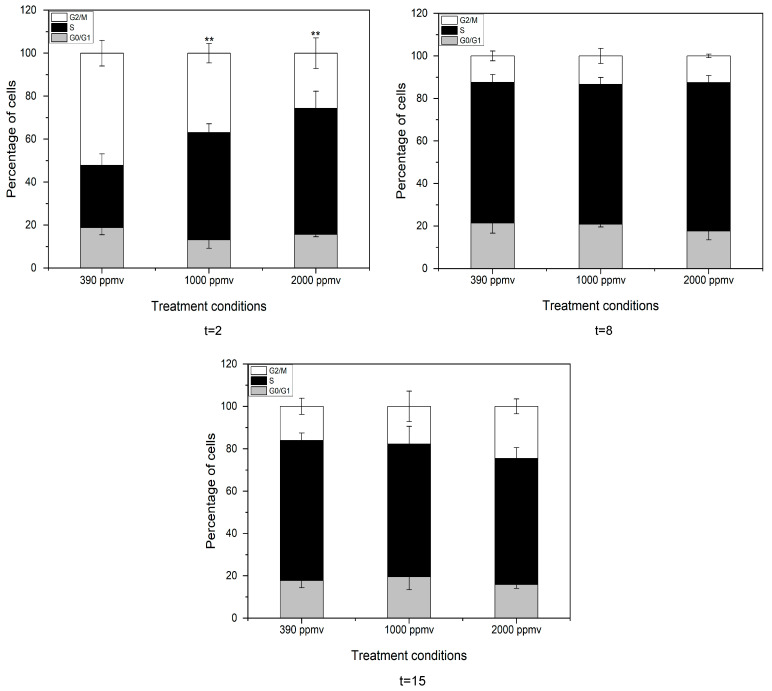
Cell cycle distribution of *K. mikimotoi* exposed to different *p*CO_2_ levels. “**”: *p* < 0.01. Note: The cells was divided into three groups by flow cytometry: Phase G_0_/G_1_, S and G_2_/M cells, The DNA content of G_1_/G_0_ phase cells: diploid cells (2n); the G_2_/M phase: tetraploid cells (4n); S phase: between diploid and tetraploid (2n-4n).

The alteration of the cell cycle was analyzed by FCM (Figure 4). The percentage of cells in the G_2_ phase decreased from approximately 52.15% to 25.7% while those in S phase significantly increased from 29.08% to 58.67% (*p* < 0.05) with the increase of *p*CO_2_ in the first two days after exposure (*p* < 0.05). However, recovery was appeared in the alteration of the cell cycle after e days of exposure. Thereafter, there was no significant difference in cell cycle changes between the final treatment groups and the control group. It seemed that cell cycle arrest in S phase occurred at the initial period of acidifying stress, but prolonging the exposure time would alleviate or even eliminate this negative impact.

### 2.5. Discrimination of H^+^ and Carbon System Alterations in Apoptosis and the Cell Cycle

**Figure 5:**
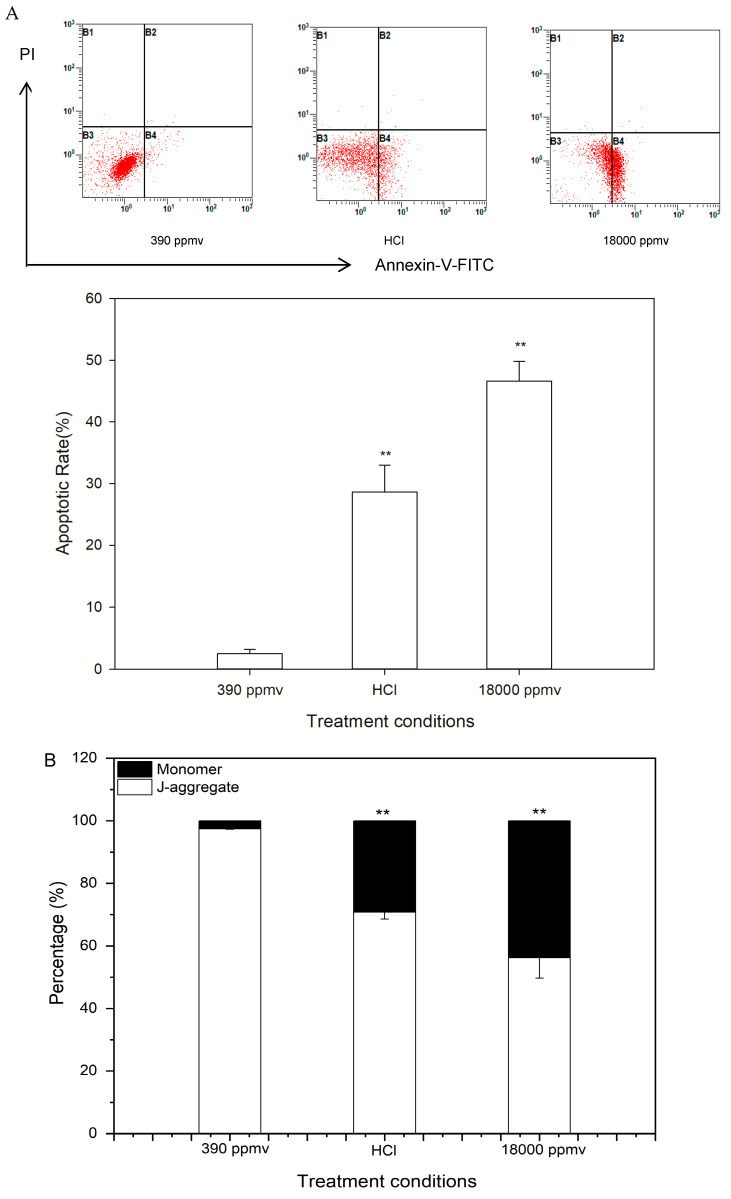

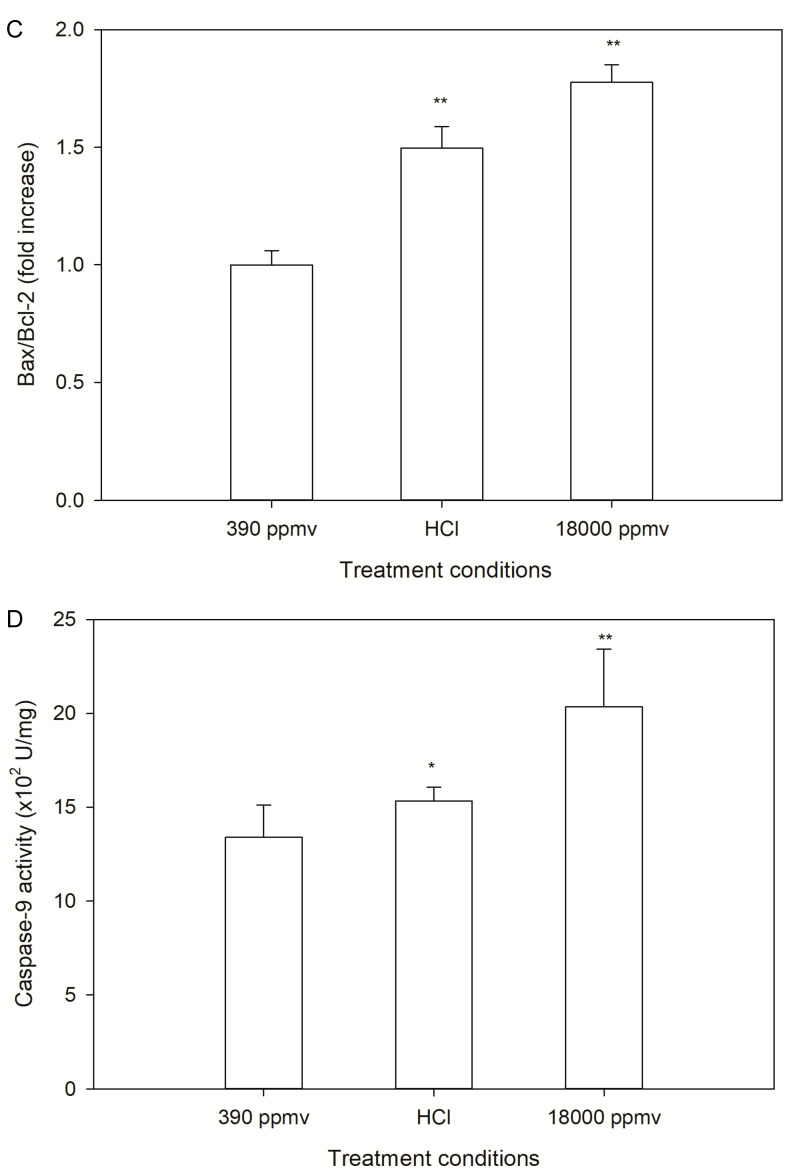

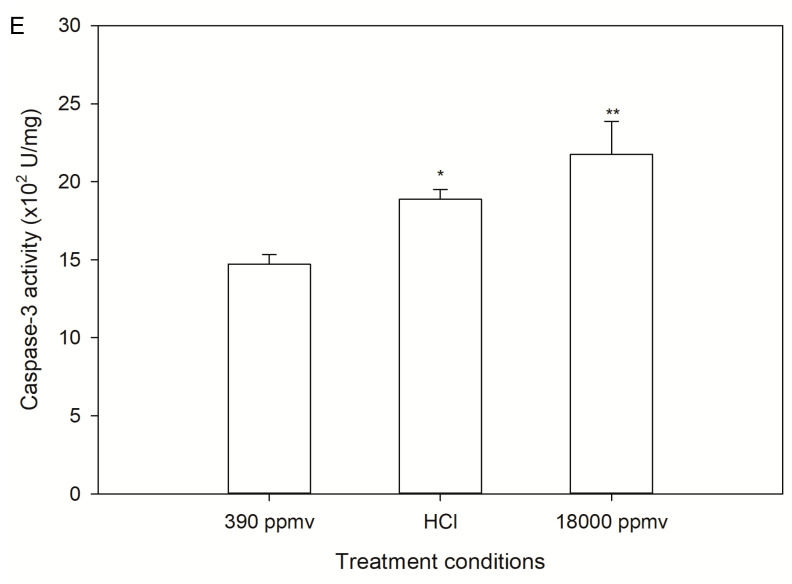
Alteration of cellular apoptosis of *K. mikimotoi* when exposed to seawater acidification caused by CO_2_ enrichment and HCl addition. (**A**) Representative graphs of cell apoptosis obtained from flow cytometry analysis and the percentage of apoptotic cells were shown by histogram. (**B**) changes of mitochondirol membrane potential (MMP): percentage of JC-1 with monomer and J-aggregate were shown by histogram; (**C**) changes of Bax/Bcl-2 ratio; (**D**) changes of Caspase-9 activity; (**E**) changes of Caspase-3 activity. * indicated the significant difference between the treatment groups with the control group at *p* < 0.05 level and ** indicated the most significant at *p* < 0.01 level, and data were expressed as means ± SD (*n* = 3). Note: the damaging level indicated from the percentage of JC-1 monomer with green fluorescence.

Based on acute toxicity experiment, a further study was performed to identify whether H^+^ or ions in the carbon system played a decisive role on the change of apoptosis and cell cycle. Cellular apoptosis of *K. mikimotoi* was induced by seawater acidification after 24 h of exposure, and the apoptotic rate greatly increased from 2.5% in the control to 28.65% and 46.6%, respectively, in the HCl addition and CO_2_ enrichment groups when the pH was 6.5 (*p* < 0.05) (Figure 5A). CO_2_ enrichment had a more noxious impact on the apoptosis of *K. mikimotoi* than that of HCl treatment at the same pH value.

The percentage of JC-1 monomer cells, which indicated mitochondrial membrane potential (MMP) damage, was found to greatly increase (by approximately 26.7% and 41.3%) in the treatment groups with a pH of 6.5 compared with that of the control (Figure 5B). Furthermore, a more obvious increase was found in the CO_2_ enrichment group, indicating that the impairment was more serious than that in the HCl treatment group.

The expression of Bax in the treatment groups with a pH level of 6.5 was significantly higher than that of Bcl-2, and Bax/Bcl-2 increased greatly, by approximately 1.5- and 1.8-fold, respectively, compared with that of the control (*p* < 0.05) (Figure 5C). The results showed that short-term (i.e., 24 h) seawater acidification activated the apoptotic pathway and further induced the apoptosis of *K. mikimotoi*, and the impact induced by CO_2_ enrichment was more serious than that induced by HCl addition.

The Caspase-3 and -9 activity in the treatment groups (HCl and 18,000 ppmv) was significantly higher than that in the control group (390 ppmv). The activity of Caspase-9 increased greatly, by approximately 1.14- and 1.46- fold higher in the treatment groups than that of the control (*p* < 0.05) (Figure 5D). A similar trend was observed for Caspase-3, which increased by approximately 1.28- and 1.48-fold (*p* < 0.05) (Figure 5E) higher in the treatment groups than that of the control. The results suggested that Caspase-9 and Caspase-3 were activated throughout the experiment by seawater acidification and the CO_2_ enrichment group was more obvious.

**Figure 6:**
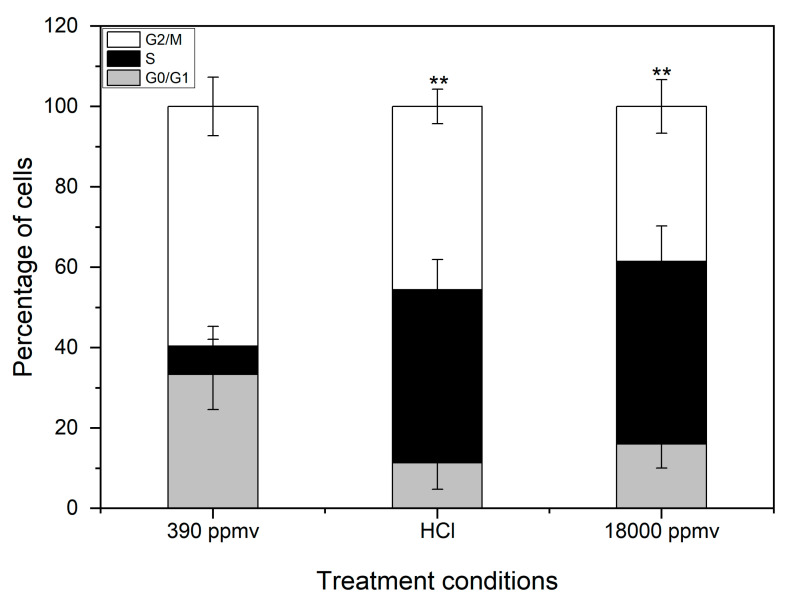
Cell cycle distribution of *K. mikimotoi* exposed to seawater acidification caused by CO_2_ enrichment and HCl addition. “**”: *p* < 0.01. Note: The cells was divided into three groups by flow cytometry: Phase G_0_/G_1_, S and G_2_/M cells, The DNA content of G_1_/G_0_ phase cells: diploid cells (2n); the G_2_/M phase: tetraploid cells (4n); S phase: between diploid and tetraploid (2n-4n).

The cell cycle distribution of *K. mikimotoi* is shown in Figure 6. After 24 h of treatment with seawater acidification, the percentage of cells in the G_2_ phase significantly decreased, from 59.65% to 45.57% and 38.51% in the HCl addition and CO_2_ enrichment groups, respectively (*p* < 0.05). In contrast, the number of cells in the S phase increased significantly (from 7.02% to 43.02% and 45.45% in the HCl addition and CO_2_ enrichment groups, respectively) compared with that of the control group (*p* < 0.05) (Figure 6). This result indicated that the CO_2_ enrichment and HCl addition partially arrested cells in the S phase, and the effect of the CO_2_ enrichment was more obvious than that of the HCl addition.

The results of the acute toxicity experiments showed that treatment with CO_2_ enrichment and HCl addition induced apoptosis in *K. mikimotoi* and partially arrested cells in the S phase, but the effect of CO_2_ enrichment was more harmful than HCl addition to *K. mikimotoi* in seawater of the same pH. This result was presumed to indicate that H^+^ was not the only factor that resulted in the impact of seawater acidification and that carbon system alteration may play an essential role in the impacts of seawater acidification.

## 3. Discussion

### 3.1. What’s the Possible Explanation for the Different Growth Modes of K. mikimotoi Exposed to Short or Long Period of Seawater Acidification

Elevated CO_2_ levels have been confirmed to stimulate the growth of many phytoplankton species, including the diatom *Pseudo nitzschia* sp [41], the raphidophyte *Heterosigma akashiwo* [42,43], and *Alexandrium fundyense* [44]. The dinoflagellate *Karlodinium veneficum* [45] also displayed abundant growth rates under increased *p*CO_2_ concentrations. We observed a slightly different phenomenon in *K. mikimotoi* with different *p*CO_2_ exposures. The specific growth rate was inhibited during the first four days after exposure compared with that of the control, while prolonging the duration time alleviated the inhibition. In other words, short-term seawater acidification inhibited the growth of *K. mikimotoi* in a clear concentration-dependent manner, while adaptation occurred with increasing time, and growth stimulation occurred within a certain time range in the exponential phase. It has been shown that short-term acidification has a negative effect on algae. This might be because sharply elevated *p*CO_2_ in seawater, together with other chemical changes, altered the periplasmic redox activity or the permeability of cellular membranes [46] and perturbed cell membrane ion channels, therefore acting as a stressor and affecting red tide algae [47]. Gazeau et al. [10] also showed that reducing carbonate ion concentrations through acidification reduced the iron uptake of phytoplankton and hypothesized that this resulted from a decreased ability of phytotransferrin, an iron binding protein, to bind with complex inorganic iron at the cell surface. However, growth differentiation was found with increased acidification time until the cells reached the exponential phase. The specific growth rate in the treatment groups increased obviously compared with that in the control, indicating the restoration of growth inhibition. The simulation results of the logistic equation provided further evidence of the observation. The carrying capacity (*K*) was almost equal to the maximum value of the cell density in the exponential phase. We also found that the peak appeared in the 2000 ppmv group. In fact, similar results have been reported. For instance, *Alexandrium* cell densities were significantly and consistently enhanced when natural populations were incubated at 150 Pa *p*CO_2_ compared with those in populations incubated at 39 Pa. During natural *Alexandrium* blooms in Northport Bay, *p*CO_2_ concentrations increased over the course of a bloom to more than 170 Pa and were highest in regions with the greatest *Alexandrium* abundances, suggesting *Alexandrium* may further exacerbate acidification and/or be especially adapted to these acidified conditions [44]. Cell proliferation was achieved through the cell cycle. In this study, we found that short-term acidification arrested microalgal cells in the S phase and prolonging the duration of exposure to acidification alleviated cellular arrest. This means that short-term acidification inhibited cell division, which was consistent with the results of many previous studies [48], while an adaptation occurred with increasing time. This was one possible explanation for the observed growth performance at the population level.

In addition, dinoflagellates have been suggested to be at a disadvantage with regard to photosynthetic carbon fixation due to the presence of low affinity forms of CO_2_-fixing enzymes, including ribulose-1, 5-bis-phosphate carboxylase-oxygenase (type II Rubisco) [49,50,51], under the present ocean conditions of low CO_2_ and high O_2_. The carbon fixation ability of dinoflagellates is said to be well below saturation at present CO_2_ levels [50,52], and thus, extremely sensitive to changes in CO_2_ concentrations. We found that Rubisco activity and the carbonic anhydrase (CA) activity of *K. mikimotoi* in the treatments were significantly decreased during the first 24 h, indicating that the reduction in photosynthesis and CCMs and the changes in seawater chemistry caused by *p*CO_2_ might be responsible for the result. However, a difference was observed on the eighth day, when Rubisco activity increased while CA decreased significantly (*p* < 0.05) in both treatments compared with that of the control, and adaptation was assumed to occur. Chen et al. [47] reported that short-term exposure to reduced pHnbs (7.70) decreased the photosynthesis and light use efficiency in algae. However, acclimation to a reduced pH level for 1~19 generations led to recovered photosynthetic activity. The results of this study were consistent with those of the abovementioned reports. Mackey et al. [53] reported that an increase in the CO_2_ concentration could potentially decrease the need for CCMs (e.g., by acquiring CO_2_ via diffusive uptake rather than active pumping of HCO_3_^−^ or reducing the requirement to express CAs), and this downregulation could potentially allow for energy and resources to be allocated to other physiological processes, such as the assimilation of other nutrients, leading to stimulated growth and photosynthesis [16,54,55]. In fact, these characteristics of adaptation to seawater acidification are one of the causative reasons for dinoflagellate bloom formation. Some documents have suggested that HABs easily develop in acidified environments. *Vicicitus globosus*, for instance, had a selective advantage under ocean acidification, increasing its abundance in natural plankton communities at CO_2_ levels higher than 600 μatm and developing blooms above 800 μatm CO_2_ [25]. HABs have increased over the past half-century [39,56], and the levels of atmospheric and surface water CO_2_ concentrations have concurrently increased by more than 25% [57]. Changing levels of dissolved inorganic carbon in surface waters may impact phytoplankton inorganic carbon fixation [16,58]. Thus, rising CO_2_ concentrations in surface waters may potentially contribute to the global expansion of HABs (i.e., the fertilization effect) [59,60]. On the other hand, the increase of CO_2_ or decrease of pH have been proven to alleviate this carbon limitation and reduce the need for energetically taxing CCMs, and the resultant shift in competitive balance may alter the phytoplankton community composition [50,61]. In other words, some dinoflagellates might benefit from decreasing pH because of their wide diversity of CCM efficiencies [62]. Previous studies have also found carbon fixation rates generally mirrored the trends of toxicity. One plausible explanation is that enhanced toxin synthesis could be due to an overall enhanced metabolic activity, and elevated *p*CO_2_ could be the catalyst for increased amounts of energy and carbon needed for phytotoxin production [63]. Likewise, *Karlodinium veneficum*, an ichthyotoxic dinoflagellate, has been shown to increase growth rates and produce more carbon-based karlotoxin under elevated levels of *p*CO_2_ [45]. In a similar study, increased growth of *A. tamarense* was seen, as the photosynthetic properties also showed a positive growth response when exposed to higher *p*CO_2_. Increased *p*CO_2_ also induced an increase in cellular toxin production in the dinoflagellate *A. tamarense* [64]. It can be inferred that *K. mikimotoi* was not only significantly increased in growth and photosynthetic carbon fixation, but also increased its toxicity when exposed to high *p*CO_2_, which may further enhance the allelopathy of *K. mikimotoi* to other species, resulting in the competitive potential of toxic HAB species as ocean acidification intensifies. *K. mikimotoi* is one of the most causative bloom-forming species in coastal China and is usually a dominant species. The adaptive mechanism of photosynthetic carbon fixation is thus assumed to explain this phenomenon.

We observed microalgal apoptosis within 24 h. The elevated *p*CO_2_ increased the Bax/Bcl-2 ratio and magnified apoptosis signaling by transferring extracellular apoptotic signaling to the apoptosis pathway. The simultaneous impairment of the mitochondrial membrane activated Caspase-9 and its downstream effector Caspase-3 trigger apoptosis. An interaction was observed between cellular apoptosis and the change in cell density during the first 24 h, and apoptosis was thought to be the adaptative response to the elevated *p*CO_2_. A hypothesis called the “altruistic adaptation theory” was proposed, the main content of which was that damaged cells could be removed from a population of single-celled organisms by programmed cell death, reducing the burden on the surviving cells to the benefit of the whole group [65]. The inflection point from negative to positive effects of elevated CO_2_ was affected by the degree of acclimation to acidification. Again, on the eighth day after exposure, apoptosis in the treatment groups was no longer observed. The results further demonstrated the speculation that *K. mikimotoi* had undergone adaptive changes under long-term exposure to CO_2_-induced seawater acidification. Interestingly, the rates of apoptosis in the 2000 ppmv and 1000 ppmv groups were significantly reduced on the 15th day after treatment, and the abovementioned indicators showed a downward trend compared with those of the control group. Some studies have suggested that high CO_2_ treatments allow cells to potentially downregulate their carbon concentrating mechanisms (CCMs), which saves up to 20% of CCM-related energy expenditures [66,67]. This would mean that high CO_2_ would raise the energy use efficiency and excessive energy dissipation strategy of *K. mikimotoi*. We might assume that *K. mikimotoi* cells reallocate intercellular energy to adapt to the new environment and that more energy is used to extend cell life. Previous studies have proven that, while *K. mikimotoi* has a wide range of suitable pH values for growth, values of 7.2~9.2 can maintain faster growth. This was consistent with our study, which found that *K. mikimotoi* has the ability to acclimate to the expected rise in atmospheric CO_2_, up to 2000 ppmv. The red tide alga *K. mikimotoi* can increase its tolerance to decreased pH values and its competitiveness in phytoplankton communities after it has acclimated to CO_2_-induced seawater acidification. This is the main reason for the frequent bloom of harmful red tides, such as those caused by *K. mikimotoi*, in environments in which seawater acidification is intensified, and the strong adaptability of harmful algae is pivotal.

### 3.2. What’s the Possible Explanation Leading to the Different Physiological Responses of K. mikimotoi When Exposed to HCl or CO_2_-Induced Acidifying Conditions?

There were ions in the acidified seawater that seemed even more noxious than H^+^ to *K. mikimotoi*. The reduced pH level resulting from the different treatments (HCl addition and high CO_2_ concentration enrichment) led to large differences in the microalgal apoptotic rates and cell cycle, and the impacts induced by the CO_2_ treatment were more severe than those induced by the HCl treatment in seawater of the same pH. We speculated that the effects of seawater acidification caused by a high CO_2_ concentration on *K. mikimotoi* were not dominated by an increase in H^+^ concentration and that the effects of other factors related to CO_2_ were even more pronounced. Previous studies have suggested that the toxicity of CO_2_ might be largely underestimated compared with that of H^+^. Elevated seawater CO_2_ concentrations can affect the functioning of molecules, cells, tissues, and whole organisms through factors such as CO_2_ itself as well as CO_2_-related changes in pH and bicarbonate levels [68,69]. Moreover, Xu et al. [70] reported that acidification interferes with the processes of ingestion and digestion in *Mytilus edulis*. However, the negative impacts induced in the CO_2_ group were more severe than those induced in the HCl group. This was consistent with the results of our study. Altogether, these results provide further evidence that the impact of seawater acidification on *K. mikimotoi* is based on factors related to CO_2_, not just H^+^.

## 4. Conclusions

Short-term seawater acidification inhibited the growth of *K. mikimotoi*, and the combination of inhibition of photosynthetic carbon fixation, cell cycle arrest, and apoptosis were the possible causes. However, prolonged exposure would induce the adaptive responses, presented by the recovery or even stimulation of the algal growth. Ions dissociated by CO_2_ dissolution other than H^+^ itself were more important to the observation. Results might elucidate the dominant role of *K. mikimotoi* during the occurrence and throughout the duration of blooms in the East China Sea.

## 5. Materials and Methods

### 5.1. Microalgal Cultivation

*K. mikimotoi* was obtained from the Algal Culture of Fisheries College Collection at the Ocean University of China. A batch cultivation was performed in the present study, and cells were cultured in f/2 medium [71] at 20 ± 1 °C with a photon flux density of 75 μmol photon m^−2^ s^−1^. The light-dark cycle was set at 12:12 h. Cells in the exponential phase were used for the experiments.

### 5.2. Acidifying System Set-Up and Experimental Design

Two level of acidification were applied, namely 1000 ppmv (pH 7.8; the prediction of atmospheric CO_2_ level in 2100) and 2000 ppmv (pH 7.5; the prediction of atmospheric CO_2_ level in 2300) based on our previous studies [38,72]. *p*CO_2_ of 390 ppmv (pH 8.1; ambient seawater pH) was used as the control. The *p*CO_2_ alteration was achieved in the experimental system with gentle bubbling of 0.22 μm-filtered ambient air and air/CO_2_ mixtures, which were generated by CO_2_ light incubator (HP400G-D, Ruihua Instrument & Equipment Ltd., Wuhan, China), with a variation of less than 5%. The pH values used during the experiment were determined using a pH meter (Seven Compact™ S210k, Mettler Toledo, Switzerland), which was calibrated daily with a standard buffer system from the National Bureau of Standards (NBS). The salinity was measured daily using a handheld salinometer (WY028Y, Huarui, China). All other carbonate system variations were calculated using CO_2_SYS software according to the method of Lewis et al. [73].

The sterile seawater was divided into 3 groups, and 3 different CO_2_ treatments (390, 1000 and 2000 ppmv) were performed 24 h before microalgal inoculation to allow the carbonate system to stabilize. Then, cells in the exponential phase were inoculated into 500 mL flasks with 300 mL acidified seawater enriched with f/2 medium, and the initial cell density was adjusted to 1 × 10^4^ cells mL^−1^. To maintain consistent CO_2_ in the cultures during the experiments, the cultures were continuously aerated by air and two different CO_2_ concentrations (1000 and 2000 ppmv) controlled by the incubator through the experiment. The cultivation was conducted for 15 days thereafter, and measurements were conducted on 2, 8, and 15 days after exposure.

### 5.3. Population Dynamics

Breifly, samples of 1 mL of the microalgal solution from each treatment were fixed every two days with acidic Lugol’s and the cell density was determined according to the method of Guillard [74].

The specific growth rate (*μ*) was calculated using the following equation:

$$\mu =(InN_{j}-InN_{i})/(t_{j}-t_{i})$$

where *N_j_* and *N_i_* represent the average cell numbers at times *t_j_* and *t_i_*, respectively.

A logistic equation was applied to quantitatively characterize the growth performance in the different *p*CO_2_ treatments.

### 5.4. Determination of Photosynthetic Carbon Fixation

Rubisco and carbonic anhydrase (CA) are two key enzymes that determine the rate of carbon assimilation in the dark reaction of photosynthesis. Their enzymatic activities were spectrophotometrically determined using ELISA kits (Shanghai Jining Shiye, Shanghai, China) according to the manufacturer’s protocol, and the results were expressed as U mg^−1^ protein. The soluble protein content was measured using the Bradford method [75].

### 5.5. Estimation of the Key Index in the Apoptotic Pathway

(1) Cellular apoptotic rate (Annexin V-FITC/PI)

The cell apoptotic rate was detected using the Annexin V-fluorescein isothiocyanate (FITC)/propidium iodide (PI) double staining method by flow cytometry (FCM). Cells in each treatment were collected on the same day and washed twice with cold phosphate-buffered saline (PBS, pH 7.2–7.4). The staining was performed using the Annexin V-FITC double staining kit (BD Pharmingen, San Diego, CA, USA) at 26 °C in the dark for 15 min, and the PI was added thereafter. The abovementioned prepared solutions were analyzed with the FL-1 channel for FITC fluorescence and the FL-3 channel for PI fluorescence using an FC 500 MPL flow cytometer (Beckman Coulter, CA, USA). The apoptotic rate was indicated by the percentage of early apoptotic cells that were stained by Annexin V-FITC.

(2) Mitochondrial membrane potential (MMP) changes

The mitochondrial membrane potential (MMP) was detected by FCM, which indicated the percentage of monomers and J-aggregates (JC-1, Beyotime Bio. Inc., Nantong, China). JC-1 probes, at a concentration of 6 μM, were added to the culture medium and incubated for 20 min at 26 °C, as described by the manufacturer. MMP changes were estimated using FCM equipped with FL-1 and FL-3 channels for monomers and J-aggregates (Beckman Coulter, Brea, CA, USA), and the MMP was indicated by the percentage of JC-1 with monomers and J-aggregates.

(3) Changes in the Bax/Bcl-2 ratio

Bax is a pro-apoptotic protein and Bcl-2 is an anti-apoptotic protein. The elevation of its ratio indicated the increase of cell apoptosis. The content of Bax and Bcl-2 was measured in the microplate using ELISA kits (Shanghai Jining Shiye, Shanghai, China) according to the manufacturer’s instructions. The absorbance (OD) of Bax and Bcl-2 was measured at 450 nm using a Perkin-Elmer microplate reader, and the content of Bax and Bcl-2 in the samples was calculated based on the standard curve.

(4) Activities of Caspase-9 and Caspase-3

The activity of Caspase-9 and Caspase-3 was measured using colorimetric activity assay kits (Beyotime Bio. Inc., Nantong, China) according to the manufacturer’s instructions and the descriptions found in our previous studies [48,76], and the activity level was recorded as U mg^−1^ protein.

### 5.6. Determination of the Cell Cycle

Propidium iodide (PI), a stain that can be embedded in double-stranded DNA, produces red fluorescence, and the fluorescence intensity is proportional to the DNA content. Microalgal cells used for the analysis of the cell cycle were prepared according to the method of propidium staining (Beyotime Bio. Inc., Nantong, China). The treated cells were incubated for 30 min at room temperature and thereafter were analyzed using the FL-3 channel for PI fluorescence. The DNA content was determined simultaneously by FCM. The percentage of cells in the different phases of the cell cycle was determined using MultiCycle software (Beckman Coulter).

### 5.7. Discrimination of H^+^ and Ions in Carbon System Alteration on Apoptosis and Cell Cycle Based on Acute Toxicity Experiment

Geological CO_2_ sequestration was a practical tool in reducing emissions [77], however, it is accepted that sub-seabed storage leaks are possible over time [78]. These leaks could cause severe reductions in pH (even achieved pH 5.6). Therefore, pH 6.5 was used as an acute toxicity test to identify the impacts of H^+^ and ions in the carbon system on the observed results by using HCl addition and CO_2_ enrichment. A pH value at extreme acidifying condition of pH 6.5 (about 18,000 ppmv) was simulated by 1 M HCl or CO_2_ mixed gas. Cellular growth and apoptosis were analyzed on the second day after exposure according to the description in 2.6 and 2.7 without other description.

### 5.8. Statistical Analysis

Analysis of variance was carried out using SPSS 16.0. All experiments were performed at least three times. The results are expressed as the mean ± standard deviation (SD), and one-way analysis of variance was used for comparisons between groups. Values of *p* < 0.05 were considered statistically significant.

## Figures and Tables

**Table 1:** Simulation on population dynamics with different *p*CO_2_ levels by logistic equation.

Groups	Equation: $N_{t}\;=\;K/1+e^{a-rt}$			
	*K* (ind mL^−1^)	*a*	*r* (d^−1^)	*Tp* (d)
390 ppmv	177.69 ± 5.63	7.10 ± 0.22	0.44 ± 0.02	16.14 ± 0.23
1000 ppmv	188.45 ± 5.07	6.82 ± 0.20	0.46 ± 0.03	14.85 ± 0.53
2000 ppmv	178.37 ± 2.97	6.68 ± 0.13	0.46 ± 0.02	14.53 ± 0.35

*r*: the maximum instantaneous growth rate, *K*: the carrying capacity, *Tp*: inflection point of population growth curve.

## Data Availability

Data are contained within the article.
